# What's About Adolescent Online Gamers' Mental Health? Psychological and Cortisol‐Stress‐System Outcomes in Childhood and Adolescence: A Prospective Longitudinal Case‐Control Study

**DOI:** 10.1002/hsr2.71135

**Published:** 2025-08-20

**Authors:** Stefan Mestermann, Peter A. Fasching, Matthias W. Beckmann, Jonas Hemetsberger, Oliver Kratz, Gunther H. Moll, Maximilian Bailer, Johannes Kornhuber, Bernd Lenz, Anna Eichler, the IMAC‐Mind Consortium, Rainer Thomasius, Nicolas Arnaud, Tobias Banaschewski, Herta Flor, Frauke Nees, Johannes Kornhuber, Michael Klein, Olaf Reis, Tanja Legenbauer, Antonia Zapf

**Affiliations:** ^1^ Department of Child and Adolescent Mental Health University Hospital Erlangen, Friedrich‐Alexander‐Universität Erlangen‐Nürnberg (FAU) Erlangen Germany; ^2^ Department of Obstetrics and Gynaecology University Hospital Erlangen, Friedrich‐Alexander‐Universität Erlangen‐Nürnberg (FAU) Erlangen Germany; ^3^ Department of Psychiatry and Psychotherapy University Hospital Erlangen, Friedrich‐Alexander‐Universität Erlangen‐Nürnberg (FAU) Erlangen Germany; ^4^ Department of Addictive Behavior and Addiction Medicine, Central Institute of Mental Health, Medical Faculty Mannheim Heidelberg University Mannheim Germany; ^5^ University Medical Center Hamburg‐Eppendorf Hamburg Germany; ^6^ Central Institute of Mental Health Mannheim Germany; ^7^ University Medical Center Schleswig Holstein Kiel University Kiel Germany; ^8^ Catholic University of Applied Sciences Cologne Germany; ^9^ University Medicine of Rostock Rostock Germany; ^10^ Ruhr‐University Bochum Bochum Germany

**Keywords:** adolescent mental health, biological stress, developmental psychology, hair cortisol, internet gaming disorder, online gaming, parental influence

## Abstract

**Background and Aims:**

Internet gaming disorder (IGD) is a relevant psychiatric disorder during adolescence. Excessive digital gaming has been associated with youth mental health problems. Prospective studies combining psychological and biological outcomes in childhood and adolescence are rare.

**Methods:**

A sample of *n* = 178 children was studied at primary school age (*M* = 7.7 years, t1) and during adolescence (*M* = 13.3 years, t2). Data was collected in mother‐ (t1, t2) and self‐reports (t2) on screen time, gaming behavior, emotional/behavioral symptoms and biological/psychological stress. Adolescent gamers and nongamers were compared using sex‐controlled (co)variance analyses and correlations with the gaming scores were carried out.

**Results:**

At t1, there were no differences between adolescent gamers versus nongamers. At t2, self‐reports indicated elevated psychological stress (*p* = 0.01) and an increase in behavioral symptoms (*p* = 0.01) in both sexes. Furthermore, gaming girls exhibited a higher prevalence of self‐reported emotional symptoms (*p* = 0.05); no differences were observed in mother‐reports or hair cortisol concentrations. In the gaming group, more DSM‐5 internet gaming disorder (IGD) symptoms at t2 were associated with higher mother‐rated total symptoms and digital gaming screen time at t1 in boys (*p* = 0.03/0.04), higher self‐rated psychological stress and more emotional/behavioral/total symptoms in boys (*p* = 0.002–0.02) and lower hair cortisol concentrations in girls (*p* = 0.003) at t2.

**Conclusion:**

The phenomenon of online gaming in adolescence had no precursors in childhood, while it was associated with current mental health and stress parameters. The extent of gaming, as defined by the DSM‐5 IGD symptoms, was found to be associated with screen time and mental health issues in childhood and adolescence. Consequently, it can be hypothesized that adolescent online gaming is a symptom of earlier as well as a risk factor for current mental health problems during adolescence, with specific consequences for boys and girls.

## 1 Introduction

**Figure 1:**
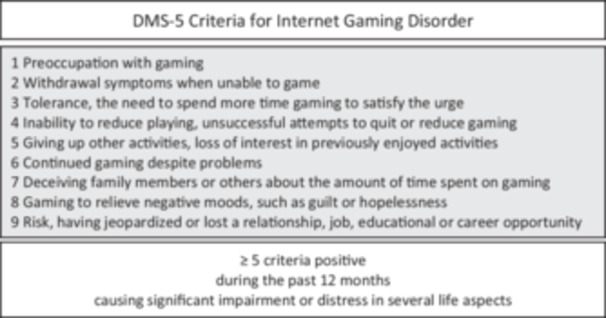
DSM‐5 criteria for internet gaming disorder [14].

Digital gaming has become a prevalent form of leisure activity among children and adolescents. In developed countries, the majority of children and adolescents, predominantly male (with a male‐to‐female ratio approximately twice as high), engages in regular, daily video gaming [1, 2]. Recent studies have indicated that early adolescents are spending an increasing amount of time engaged with screens and using social media; this rise has been particularly pronounced since the onset of the COVID‐19 pandemic [3, 4]. A decline in the prevalence of games designed for offline single‐player use has been observed, while an increase in the popularity of online games, predominantly in multiplayer format, has been demonstrated [5]. Internet gaming can be regarded as a form of leisure activity; however, the potential for problematic engagement, including addiction, is a significant concern [6, 7]. Particularly for adolescents, Role Play Games (RPG) seem to bear the highest risk of IGD. This can be attributed, at least in part, to the time‐consuming nature of looting and reward systems, as well as character levelling mechanisms, which serve to perpetuate the engagement of players within the game [8, 9]. Research indicates that boys are more likely than girls to report higher screen time and more problematic video game usage [10]. Mental health concerns related to online gaming have emerged as a prominent subject in the field of of current child and adolescent psychiatric research [11]. A mounting body of research has emerged in recent years that suggests a correlation between higher levels of gaming and poor mental health outcomes, social behavior deficits and the development of psychiatric disorders, including depression, anxiety and attention deficit hyperactivity disorder (ADHD) [3, 12, 13]. In 2013, the fifth edition of the Diagnostic and Statistical Manual of Mental Disorders (DSM‐5) defined the Internet Gaming Disorder (IGD) with nine diagnostic criteria derived from psychiatric addiction disorder characteristics (Figure 1) [14, 15]. Research indicates a rising prevalence of IGD among young people, with estimates reaching up to 10% [16, 17].

When investigating the antecedents and consequences of pathological online gaming, at least two hypotheses can be proposed. Firstly, excessive gaming has been identified as a risk factor for mental health issues during adolescence. In prior studies, frequent and lengthy computer gaming was associated with child emotional and behavioral problems [18, 19, 20, 21] as well as altered psychological and biological stress‐reactions [22, 23]. Current reviews summarize these cross‐sectional associations [3, 13]. The most recent study focusing on students indicated that sleep disturbances resulting from excessive gaming may contribute to the development of mental health problems [24]. Secondly, excessive gaming may function as a secondary symptom and can be comorbid with pre‐existing mental health conditions [12]. In this context, some authors have posited that IGD may be conceptualized as a symptom of an underlying mental disorder rather than as an independent diagnosis. Excessive gaming has been identified as a dysfunctional coping strategy (“refuge“) in response to internalizing and externalizing problems. In studies conducted on childhood and adolescence populations, this hypothesis predominantly pertains to children and adolescents diagnosed with ADHD, with particular emphasis on their impaired executive function [25]. Such impaired function can lead to increased impulsivity and reduced self‐control, which may be associated with extended gaming duration [26, 27]. In studies conducted on adult populations, the presence of other mental illnesses, including mood disorders [28], social anxiety [29], and substance use [30], appears to increase the likehood of developing IGD.

To simulanteously investigate perspectives within a single research design, it is essential to conduct prospective longitudinal studies. To the best of our knowledge, no existing studies have yet examined the childhood mental health antecedents of later adolescent gaming behavior using a longitudinal repeated measures design, nor have they included hair cortisol concentration as a biological marker of stress. The present study aims to differentiate between adolescent gamers and nongamers and to establish an association between the number of DSM‐5 IGD symptoms within the gaming group in two distinct terms. Firstly, the association will be examined in relation to childhood mental health problems, with the hypothesis of adolescent gaming being a secondary symptom of pre‐existing mental health problems. Secondly, the association will be investigated in terms of current mental health problems, positing that adolescent gaming could function as a risk factor for mental health problems. The following research questions will be addressed (separately for boys and girls):

With focus on the **secondary‐symptom‐hypothesis**: Did adolescents who game online today already show more emotional/behavioral problems and higher screen time *previously* (at primary school age) than adolescents who do not game online today? Are there correlations between the number of DSM‐5 IGD symptoms in adolescence with childhood emotional/behavioral symptoms, and the amount of screen time during childhood?

With focus on the **risk‐factor‐hypothesis**: Do adolescents who game online today *currently* show more emotional/behavioral problems and more psychological and biological (hair cortisol concentration) stress than adolescents who do not game online today? Are there correlations between the number of DSM‐5 IGD symptoms in adolescence with adolescence emotional/behavioral symptoms, and psychological and biological (hair cortisol concentration) stress?

## 2 Materials and Methods

### 2.1 Study Design and Sample Characteristics

The present study uses a prospective longitudinal case‐control design with measurements in childhood (t1) and adolescence (t2), with mothers (t1, t2) and adolescents (t2) providing information regarding emotional/behavioral and stress symptoms. Hair cortisol concentrations are also measured and analyzed as a biological stress marker. The present study used data from the Franconian Cognition and Emotion Studies (FRANCES) [31, 32, 33]. A total of *n* = 188 mother‐child dyads during their primary school age (6–9 years; t1, FRANCES I) with longitudinal adolescent data (12–14 years; t2, FRANCES II) were available. Of these, *n* = 182 answered the questionnaire on DSM‐5 IGD symptoms at t2. Two twin pairs (*n* = 4) were excluded due to nonindependent measures. Finally, the present study comprised a total of *n* = 178 adolescents (age: *M*_t2_ = 13.28 years, SD = 0.31, range 12.8–14.4 years, 89/50% boys). Sociodemographic data was assessed during in person meetings with the mothers and children/adolescent (see Table 1). Additionally, weight and height were measured to calculate child body mass index (BMI). The present study encountered missing data for the following variables: *n* = 14 screen time mother‐ratings at t1, *n* = 2 psychological stress self‐ratings at t2, *n* = 1 emotional/behavioral/total symptoms mother‐rating at t1 and t2, and *n* = 58 hair cortisol concentrations (HCC) due to nongiven hair strains, medication use, high coefficient of variation CV > 0.20, or outliers > 3.29 SD.

**Table 1:** Sample characteristics.

		Primary school age t1		Adolescence t2	
		*M* (SD)/*n* (%)	Range	*M* (SD)/*n* (%)	Range
**Children**					
Age [y]		7.7 (0.76)	6.0–9.9	13.28 (0.31)	12.8–14.4
Sex	Female	89 (50)		89 (50)	
	Male	89 (50)		89 (50)	
**Mothers**					
Age [y]		40.5 (4.4)	28.0–51.0	46.1 (4.4)	33.2–56.8
Marital status	Single	24 (13.5)		11 (6.2)	
	Married	150 (84.2)		145 (81.5)	
	Divorced	1 (0.6)		20 (11.2)	
	Widowed	0 (0)		1 (0.6)	
	Missing	3 (1.7)		1 (0.6)	
Partnership	Yes	161 (90.4)		142 (79.8)	
	No	14 (7.9)		19 (10.7)	
	Missing	3 (1.7)		17 (9.5)	
Years of school attendance	> 12	103 (57.9)		108 (60.1)	
	≤ 12	74 (41.6)		70 (39.3)	
	Missing	1 (0.6)		1 (0.6)	
Family income (monthly, net)	1.000–2.000 €	15 (8.4)		9 (5.1)	
	2.000–3.000 €	36 (20.2)		23 (12.9)	
	3.000–4.000 €	38 (21.3)		35 (19.7)	
	4.000–5.000 €	36 (20.2)		36 (20.2)	
	> 5.000 €	52 (29.2)		74 (41.6)	
	Missing	1 (0.6)		1 (0.6)	

Abbreviations: M, mean value; *n*, number of participants; SD, standard deviation; %, percentages.

**Figure 2:**
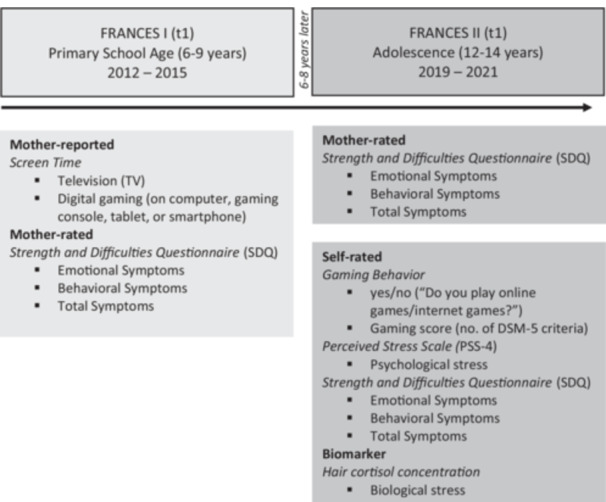
Franconian Cognition and Emotion Studies (FRANCES) design.

At t1, the participating mothers were requested to respond to a standardized questionnaire regarding their children's screen time. At t2, adolescents completed self‐report measures of emotional/behavioral/general symptoms and psychological stress via standardized questionnaires (pen‐and‐paper format), and their 1‐month cumulative HCC was measured from a 1 cm hair strand, serving as an objective biological marker of stress. At t1 and t2, maternal reports regarding the child's emotional/behavioral/total symptoms were collected using a standardized questionnaire. As illustrated in Figure 2, the study design and the variables assessed are shown. The study was approved by the Local Ethics Committee of the Friedrich‐Alexander‐University Erlangen‐Nürnberg, Germany (4596 and 353_18B) and was conducted in accordance with the Declaration of Helsinki. All mothers provided informed consent, all children informed assent.

### 2.2 Measures

The operationalization of **gaming behavior** was achieved through two distinct methodologies. At t2, to differentiate between those who engage in gaming and those who do not (**gaming vs. nongaming**), adolescents were asked on a nominal scale (yes/no) whether they play online/internet games [“*Do you play online games/internet games (e.g. FIFA online, Minecraft online, GTA online, Clash of Clans)?*”]. Moreover, at t2, the adolescents were asked to rate whether the DSM‐5 IGD criteria applied to them on a nominal yes/no scale. The number of positive DSM‐5 IGD items was used to create a **gaming score** ranging from 0 to 9. The presence of a gaming score ≥ 5 was interpreted as an IGD diagnosis [34].

At t1, mothers reported their children's **screen time** [television (TV) and digital gaming on computers, gaming consoles, tablets, and smartphones] in hours per day.

The assessment of **emotional, behavioral and total symptoms** was carried out using the *Strengths and Difficulties Questionnaire* (SDQ) [35, 36] at t1 (mother‐rating) and at t2 (mother‐rating and child‐rating). The SDQ comprises 25 items that are answered on a 3‐point Likert‐scale, ranging from 0 (not true) to 1 (somewhat true) and 2 (certainly true). The items are summarized in five subscales (emotional problems, behavioral problems, hyperactivity/inattention, peer relation problems and prosocial behavior). Each subscale contains five items. In the present study, the subscale sum score for emotional problems (= emotional symptoms) and behavioral problems (= behavioral symptoms), which ranges from 0 to 10, and the total problems score (= total symptoms) with a range from 0 to 40 (excluding ‘prosocial behavior’ items) were calculated.

At t2, adolescent **stress** was measured in **psychological and biological** dimensions: Adolescents were asked to complete standardized self‐report measures of psychological stress using the *Perceived Stress Scale* (PSS‐4) [37, 38]. A set of four items asks about subjective stress in the past month (e.g., “In the past month, how often have you felt that you were unable to control important things in your life?”) and is rated on a 5‐point Likert scale (0 = never to 4 = very often). The total sum score, ranging from 0 to 16, has been used to depict the level of stress.

To assess the **hair cortisol concentration (HCC)** of children as a biological stress marker, a segment of hair measuring at least 1 cm in length was obtained from the posterior vertex region, in close proximity to the scalp. This segment was then wrapped in aluminum foil and stored within paper envelopes at a temperature of 4°C, where it remained until analysis. A questionnaire was administered to mothers to collect information regarding their children's chemical hair treatments, medication use, infection symptoms, and endocrine disorders within the 6 months before sampling. To determine HCC, the first proximal centimeter of each sample was analyzed (1 cm of hair corresponds to about 1 month of growth). *Cortisol/protein extraction:* Samples were processed according to the protocol published by Frisch and colleagues [39]. Briefly, each sample was incubated twice with 2.5 mL isopropanol, air dried, minced with grinding balls in a ball mill (Retsch GmbH, Haan, Germany) and weighed (sample weight = gross weight, i.e., vial, grinding balls and sample ‐ tar weight, i.e., vial and grinding balls). For cortisol extraction, a 4‐step method with alternating methanol and acetone extraction steps was applied and the methanol‐acetone supernatants were evaporated at 50°C. The resulting pellets were stored at −20°C until analysis and dissolved in 250 µL phosphate‐buffered saline immediately before analysis. *Determination of HCC/protein concentrations:* Protein concentrations were quantified using the Bradford Protein Assay (Roti‐Quant Protein quantitation assay according to Bradford, Carl Roth GmbH, Germany). Cortisol concentrations were detemined using salivary cortisol ELISA kits (RE52611; IBL International, Hamburg, Germany) following the manufacturers' instructions. Each sample was assayed in duplicate (Benchmark Plus microplate spectrophotometer, Bio‐Rad Laboratories, Hercules, CA, USA) and the mean values and coefficients of variation (CV) were computed for each duplicate. *Statistical assessment of HCC:* Each HCC was normalized to the sample weight and ln‐transformed (since standardized HCC were not normally distributed) as follows:

$$\text{Cortisol}-\text{to}-\text{weight ratio}\;\left(\frac{\text{pg}}{\text{mg}}\right)=\ln\left(\frac{\text{HCC}\;\left(\frac{\text{ng}}{\text{ml}}\right)\times 0.25\text{ml}}{\text{hair sample weight}\;\left(\text{mg}\right)}\times 1000\right)$$

**Equation 1** Calculation of Cortisol−to−weight ratio in assessment of cumulative 1‐month hair cortisol concentration (HCC).

The exclusion criteria included corticosteroid medication (*n* = 9), endocrinological diseases affecting hair growth (*n* = 1) and chemical hair treatment (*n* = 2). *N* = 2 children had an obesity score > 30 kg/m^2^. Furthermore, subjects were excluded if their CV was greater than 0.20 (*n* = 14) or if their outlier score exceeded 3.29 standard deviations (*n* = 3). Additionally, 27 adolescents did not consent to hair sampling.

### 2.3 Statistical Analysis

All analyses were performed using IBM SPSS Statistics, version 28. The figures were created using GraphPad Prism. A *p* ≤ 0.05 was considered significant in two‐tailed tests. Normal (Gaussian) distribution was evaluated via Shapiro‐Wilk‐test [40], variance homogeneity was tested with the Levene‐test [41]. First, the frequency of gaming and nongaming between boys and girls, as well as the distribution of DSM‐5 IGD diagnosis yes/no were compared. Additionally, independent *t*‐tests were employed to compare the gaming scores between boys and girls.

For hypotheses testing, two‐factorial analyses of variance [ANOVA [42]; between‐subject factors “gaming” (yes/no) and “sex” (boy/girl)] with t1 mother‐rated screen time (TV and digital gaming), and t1 mother‐rated emotional/behavioral/total symptoms as dependent measures were analyzed for main effect “gaming” and interaction effect “gaming × sex” in separate analyses. Next, two‐factorial analyses of covariance [ANCOVA [43]; between‐subject factors “gaming” (yes/no) and “sex” (boy/girl), covariate: t1 mother‐rated total symptoms] with t2 mother‐rated emotional/behavioral/total symptoms, t2 child‐rated emotional/behavioral/total symptoms and psychological stress, and biological stress marker (hair cortisol concentration) as dependent measures were analyzed for main effect “gaming” and interaction effect “gaming × sex” in separate analyses. The partial eta squared (*ɳ* ^2^) was reported as effect size measure (≥ 0.01 small, ≥ 0.06 medium, ≥ 0.14 large effect).

In dose–response analyses, within the gamers‐group, separately for boys and girls, the gaming score was associated with t1 outcomes (mother‐rated screen time, and emotional/behavioral/total symptoms) in Pearson correlations (*r*) and with t2 outcomes [mother‐rated emotional/behavioral/total symptoms, child‐rated emotional/behavioral/total symptoms and psychological stress, and biological stress (hair cortisol concentration)] in partial correlations (*r*_p_) under control of “t1 mother‐rated total symptoms”. Correlation coefficients were interpreted as: weak (> 0.10), medium (> 0.30), or strong (> 0.50).

## 3 Results

**Figure 3:**
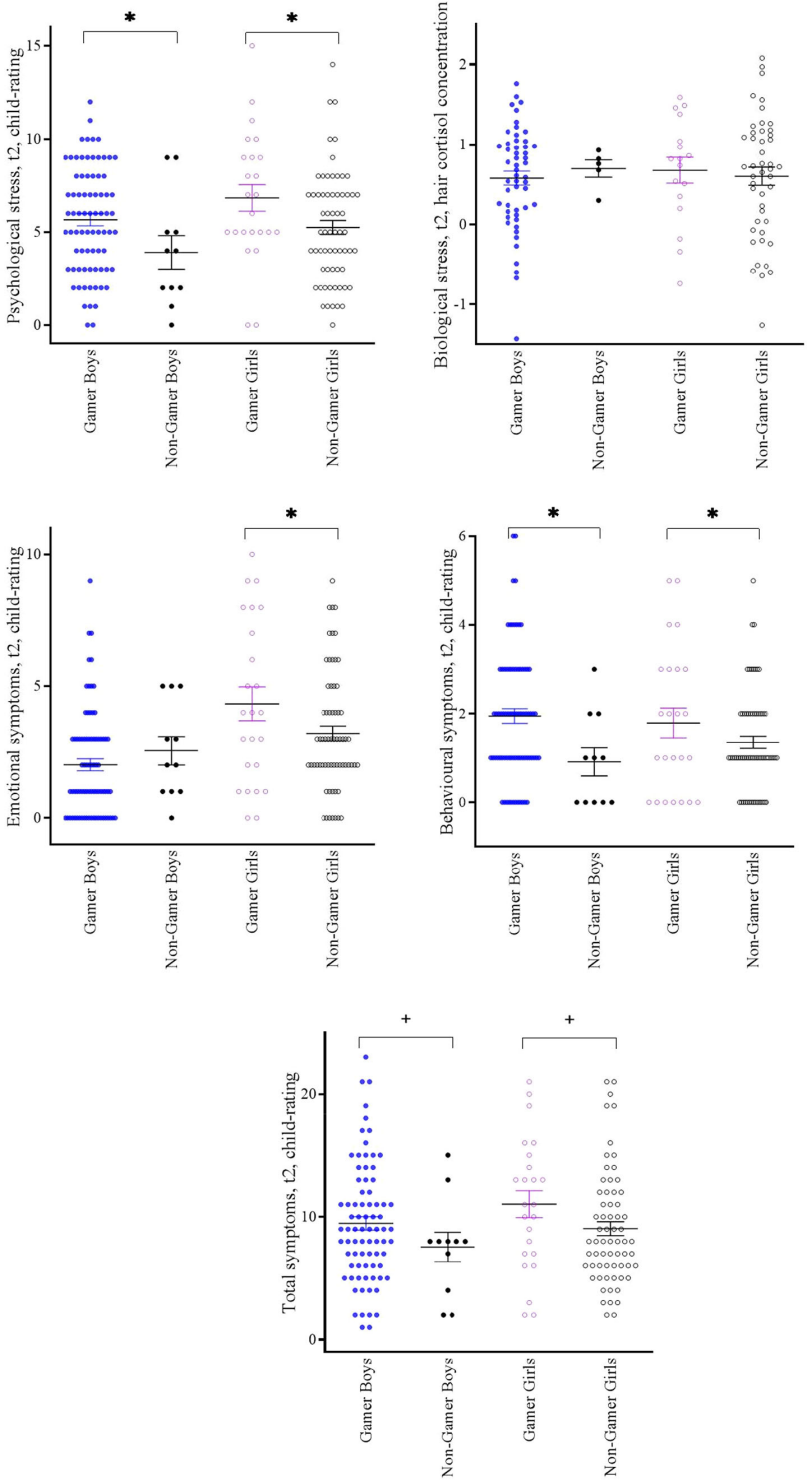
Gamers versus nongamers differences at t2 (adolescence) separately for boys and girls. Means and standard error of the means; ANCOVA results are marked with ^+^ *p* ≤ 0.10, *p ≤ 0.05. Emotional/behavioral/total symptoms: *Strengths and Difficulties Questionnaire* (SDQ). Biological stress: Hair cortisol concentration; Psychological stress: *Perceived Stress Scale* (PSS‐4).

There were *n* = 102 (76.5% boys) gaming and *n* = 76 (85.5% girls) nongaming adolescents, with a statistically significant difference between boys and girls (χ^2^ = 66.96, *p* ≤ 0.001; Table 2). In the gaming group, 4 of 78 boys (5.1%) and 0 of 24 girls (0%) met DSM‐5 criteria for IGD. The DSM‐5‐based continuous gaming scores were not significantly different between boys and girls [*T*(98) = 0.787, *p* = 0.22; Table 3]. To test hypotheses, first, in ANOVA analyses (Table 4), at primary school age (t1), there were no significant mother‐reported differences in children's screen time or emotional/behavioral/total symptoms between later adolescent gamers and nongamers (main effect *p* = 0.18–0.92, ɳ_p_^2^ = 0.000–0.01) for either boys or girls (interaction effect gaming × sex *p* = 0.07–0.91, *ɳ*_p_^2^ = 0.000–0.02). Second, in ANCOVA analyses (Table 4), at adolescence age (t2), adolescents themselves reported higher psychological stress (main effect *p* = 0.01, *ɳ*_p_^2^ = 0.04) and more behavioral symptoms (main effect *p* = 0.01, *ɳ*_p_^2^ = 0.04) for both sexes when belonging to the gamers group, and more emotional symptoms for girls (interaction effect gaming × sex *p* = 0.05, *ɳ*_p_^2^ = 0.02); post‐hoc *t*‐tests showed a gender difference within the gamers group with higher emotional symptoms in girls (*t*(100) = −3.42, *p* < 0.001). All effects were small (*ɳ*_p_^2^ = 0.02–0.04). In t2 mother‐reports, there were no significant differences between gamers and nongamers on emotional/behavioral/total symptoms (main effect *p* = 0.18–0.20, *ɳ*_p_^2^ = 0.01) for either boys or girls (interaction effect gaming × sex *p* = 0.09–0.80, *ɳ*_p_^2^ = 0.000–0.02). Hair cortisol concentrations were not associated (main effect *p* = 0.767, *ɳ*_p_^2^ = 0.001; interaction effect gaming × sex *p* = 0.523, ɳ_p_^2^ = 0.004). Figure 3 shows the AN(C)OVA results.

**Table 2:** Gaming/nongaming participants among boys/girls.

	Boys	Girls	
Gaming	78	24	102
Nongaming	11	65	76
	89	89	

*Note:* χ2 = 66.96, *p* ≤ 0.001.

**Table 3:** Descriptive data for gaming/nongaming subgroups in the total sample (*n* = 102/76) and separately for boys (*n* = 78/11) and girls (*n* = 24/65).

	Total				Boys				Girls			
	Gaming		Nongaming		Gaming		Nongaming		Gaming		Nongaming	
	*M*	SD	*M*	SD	*M*	SD	*M*	SD	*M*	SD	*M*	SD
Gaming score	1.54	1.47			1.61	1.48			1.33	1.46		
Screen Time [h/d]												
Television (TV) t1	0.83	0.69	0.66	0.49	0.84	0.74	0.68	0.53	0.79	0.54	0.66	0.49
Digital gaming t1	0.38	0.52	0.19	0.24	0.41	0.56	0.26	0.30	0.27	0.35	0.18	0.23
Stress												
Psychological t2	5.94	3.03	5.05	3.00	5.66	2.84	3.91	2.98	6.83	3.50	5.25	2.98
Biological t2	0.60	0.64	0.61	0.73	0.58	0.64	0.70	0.24	0.68	0.66	0.60	0.76
Emotional symptoms												
Mother t1	1.73	1.72	1.86	1.61	1.57	1.61	1.91	1.76	2.25	1.98	1.85	1.60
Mother t2	1.67	2.03	1.96	1.71	1.40	1.62	0.64	0.81	2.54	1.86	2.19	1.73
Child t2	2.56	2.50	3.12	2.20	2.01	2.00	2.55	1.81	4.33	3.13	3.22	2.25
Behavioral symptoms												
Mother t1	1.97	1.74	1.78	1.29	2.08	1.83	1.18	0.98	1.63	1.38	1.88	1.32
Mother t2	1.72	1.50	1.19	1.30	1.88	1.46	0.91	1.04	1.17	1.52	1.23	1.34
Child t2	1.91	1.49	1.29	1.13	1.95	1.45	0.91	1.04	1.79	1.64	1.35	1.14
Total symptoms												
Mother t1	7.63	4.42	7.20	4.50	7.73	4.59	6.09	3.39	7.33	3.90	7.38	4.65
Mother t2	7.57	4.94	6.48	4.62	7.88	4.21	4.27	2.65	5.54	6.81	6.86	4.79
Child t2	9.84	4.97	8.83	4.54	9.47	4.83	7.55	4.01	11.04	5.35	9.05	4.62

*Note:* Gaming score = Number of *DSM‐5 Internet Gaming Disorder symptoms* (range 0–9). Screen time = mother rated, hours per day. t1 = primary school age, t2 = adolescence. Emotional/behavioral/total symptoms: *Strengths and Difficulties Questionnaire* (SDQ). Biological stress: Hair cortisol concentration; Psychological stress: *Perceived Stress Scale* (PSS‐4).

Abbreviations: *M,* mean value; *n*, number of participants; SD, standard deviation.

**Table 4:** AN(C)OVA results for gaming/nongaming and boys/girls as between‐subject factors.

	Model			Main effect Gaming			Interaction effect Gaming × Sex		
	*F*	*df*/*df*	*p*	*F*	*p*	*ɳ*_p_^2^	*F*	*p*	*ɳ*_p_^2^
Screen time t1									
Television (TV)	1.06	3/160	0.37	1.30	0.26	0.008	0.01	0.91	0.000
Digital gaming	3.15	3/160	0.03*	1.85	0.18	0.011	0.081	0.78	0.001
Stress t2									
Psychological	3.91	4/170	0.005*	6.83	0.01*	0.039	0.00	0.95	0.000
Biological	0.76	4/114	0.56	0.09	0.77	0.001	0.41	0.52	0.004
Emotional symptoms t1/t2									
Mother‐rating t1	1.09	3/173	0.36	0.01	0.92	0.000	1.22	0.27	0.007
Mother‐rating t2	7.17	4/171	< 0.001**	1.67	0.20	0.010	0.07	0.80	0.000
Child‐rating t2	7.28	4/172	< 0.001**	0.26	0.61	0.002	3.97	0.05*	0.023
Behavioral symptoms t1/t2									
Mother‐rating t1	1.37	3/173	0.25	1.07	0.30	0.006	3.39	0.07+	0.019
Mother‐rating t2	8.92	4/171	< 0.001**	1.83	0.18	0.011	2.41	0.12	0.014
Child‐rating t2	3.14	4/172	0.02*	6.88	0.01*	0.038	1.01	0.32	0.006
Total symptoms t1/t2									
Mother‐rating t1	0.45	3/173	0.72	0.78	0.38	0.005	0.89	0.35	0.005
Mother‐rating t2	16.09	4/171	< 0.001**	2.07	0.15	0.012	3.01	0.09+	0.017
Child‐rating t2	4.04	4/172	0.004*	3.48	0.06+	0.020	0.08	0.78	0.000

*Note:* AN(C)OVA = Analysis of (Co)Variance. t1 = primary school age, t2 = adolescence. t1: ANOVA analyses with gaming yes/no and sex boys/girls as between‐subject factors; t2: ANCOVA analyses with gaming yes/no and sex boys/girls as between‐subject factors and “t1 mother‐rated total symptoms” as covariate. Screen time = mother rated, hours per day. Emotional/behavioral/total symptoms: *Strengths and Difficulties Questionnaire* (SDQ). Biological stress: Hair cortisol concentration; Psychological stress: *Perceived Stress Scale* (PSS‐4).

^+^ *p* ≤ 0.10.

* *p* ≤ 0.05

** *p* ≤ 0.001.

**Figure 4:**
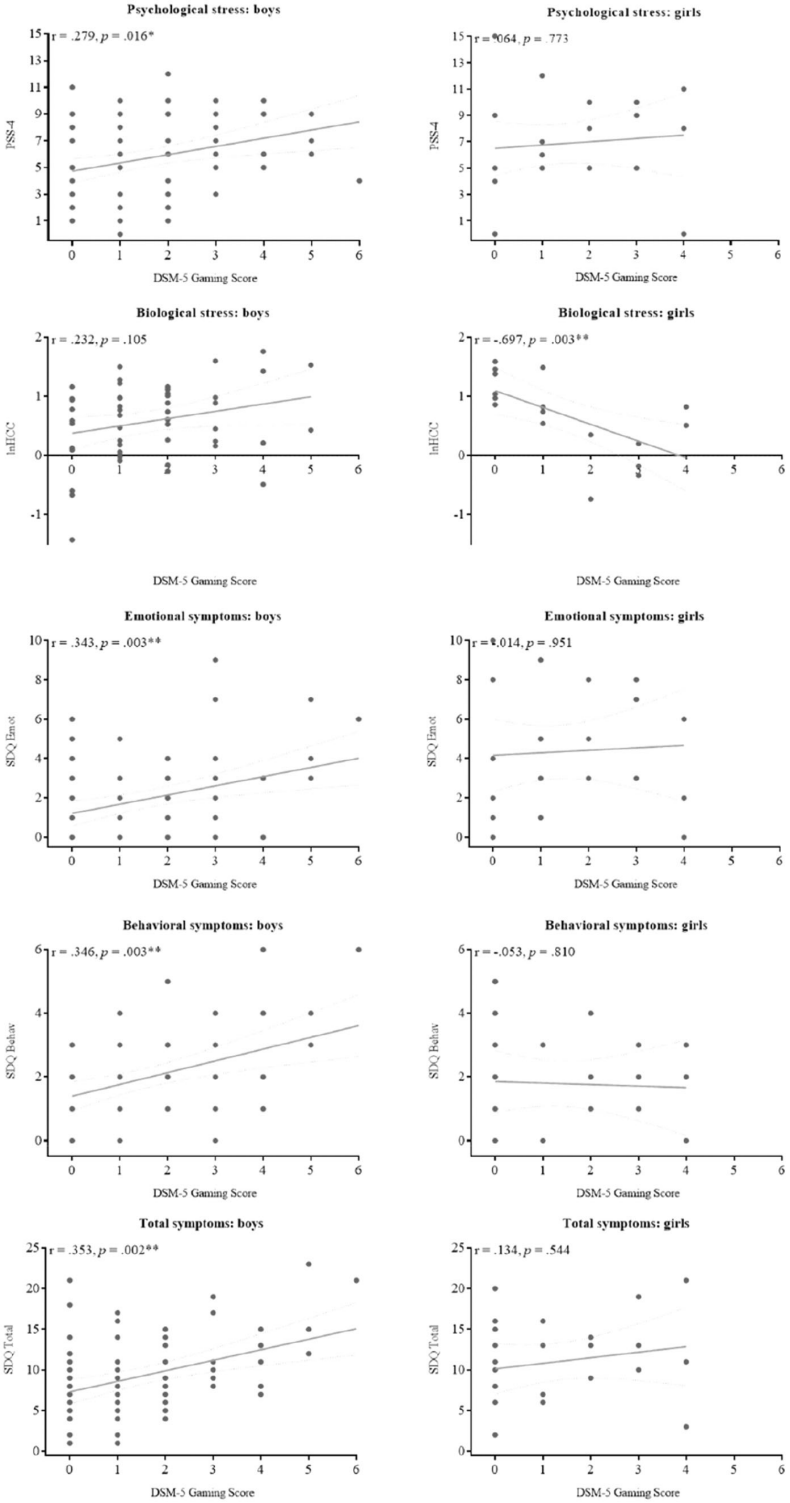
Gaming‐score correlations with t2 outcome measures separately for boys and girls. Notes: linear regression lines with standard error; partial‐correlation results are reported with *p ≤ 0.05, **p ≤ 0.001. DSM‐5 gaming score = Number of *DSM‐5 Internet Gaming Disorder symptoms* (range 0–9). Emotional/behavioral/total symptoms: *Strengths and Difficulties Questionnaire* (SDQ). Biological stress: Hair cortisol concentration; Psychological stress: *Perceived Stress Scale* (PSS‐4).

Third, within the gamers‐group, only for boys, there was a significant correlation between the t2 gaming score with t1 mother‐rated totals symptoms (*r* = 0.245, *p* = 0.03) and t1 mother‐reported digital gaming screen time (*r* = 0.241, *p* = 0.04). Other t1 variables in boys and all t1 variables in girls weren't significantly correlated with t2 gaming scores (Table 5). Within the t2 measures, only for boys, gaming scores were associated with more self‐rated psychological stress (*r*_p_ = 0.279, *p* = 0.02) and more self‐rated emotional/behavioral/total symptoms (*r*_p_ = 0.34/0.35/0.35, *p* = 0.003/0.003/0.002). Only for girls, higher gaming scores were correlated with lower hair cortisol concentrations (*r*_p_ = −0.70, *p* = 0.003). t2 partial‐correlations are visualized in Figure 4.

**Table 5:** Gaming score correlations with primary school age (t1) and adolescence (t2) outcome measures separately for boys and girls.

	Boys		Girls	
	*r*(*df*)^a^	*p*	*r*(*df*)^a^	*p*
	**t1 primary school age**			
**Screen time**				
Television (TV)	0.093	0.44	−0.198	0.38
Digital gaming	0.241	0.04*	−0.396	0.07+
**Emotional symptoms**				
Mother‐rating	0.030	0.80	0.299	0.16
**Behavioral symptoms**				
Mother‐rating	0.082	0.49	0.366	0.08+
**Total symptoms**				
Mother‐rating	0.245	0.03*	0.262	0.22
	**t2 adolescence**			
**Stress**				
Psychological	0.279 (72)	0.01*	0.064 (21)	0.77
Biological	0.232 (48)	0.11	−0.697 (14)	0.003*
**Emotional symptoms**				
Mother‐rating	0.112 (72)	0.34	0.226 (21)	0.30
Child‐rating	0.343 (72)	0.003*	−0.014 (21)	0.95
**Behavioral symptoms**				
Mother‐rating	0.194 (72)	0.35	0.365 (21)	0.09+
Child‐rating	0.346 (72)	0.003*	−0.053 (21)	0.81
**Total symptoms**				
Mother‐rating	0.008 (72)	0.95	0.211 (21)	0.33
Child‐rating	0.353 (72)	0.002*	0.134 (21)	0.54

*Note:* Gaming score = Number of DSM‐5 Internet Gaming Disorder symptoms. Screen time = mother rated, hours per day. Emotional/behavioral/total symptoms: *Strengths and Difficulties Questionnaire* (SDQ). Biological stress: Hair cortisol concentration; Psychological stress: *Perceived Stress Scale* (PSS‐4).

^a^ Degrees of freedom (df) are reported for t2 partial correlations under control of “t1 mother‐rated total symptoms.”

^+^ *p* ≤ 0.10.

* *p* ≤ 0.05.

## 4 Discussion

The present study aimed to differentiate between gaming and nongaming adolescents in terms of past (primary school age) and current (adolescence) screen time, emotional and behavioral symptoms, as well as psychological (self‐rated stress in the last month) and biological (HCC) stress measures. Furthermore, this study sought to ascertain the correlations between gaming scores, operationalized as the number of DSM‐5 IGD symptoms, and the aforementioned outcomes during primary school age and adolescence. The present study considered the IGD and other psychiatric disorders to be bidirectional variables that mutually influence each other. Addiction disorders, such as IGD, may serve as either a cause or a consequence of psychiatric comorbidities [44].

The data demonstrated that, in terms of secondary‐symptom‐hypothesis testing, adolescent gamers did not differ from adolescent nongamers on primary school age maternal ratings. The current risk factor hypothesis was tested, and adolescent gamers reported more psychological distress, behavioral symptoms, and, only for girls, emotional symptoms. These effects were small.

In the gaming group, significant associations were obseverd exclusively among boys, whereby higher adolescent gaming scores, measured as the number of DSM‐5 IGD symptoms, were associated with increased digital game screen time and elevated total symptom scores during primary school age, albeit with small effect size. Additionally, higher adolescent gaming scores were associated with greater psychological stress, also at a small effect size, and with more emotional/behavioral/overall symptoms, at a medium effect size, in boys' self‐reports. For girls, a strong effect was identified, indicating lower HCCs with higher game scores. The present study incorporated two measurement points and replicated previous studies' cross‐sectional findings associating online gaming with child/adolescent emotional and behavioral problems [18, 19, 20, 21], as well as altered psychological and biological stress responses [22, 23] in one design ‐ with different precursors and risks for boys and girls.

### 4.1 Frequency of Gaming and IGD Symptoms

This study found that 57.3% of adolescents, 87.6% of boys, and 27.0% of girls, who regularly game online, are consistent with earlier results (e.g., [1], 63% total [45]; 79% boys, 23% girls). In accordance with the findings of recent reviews and meta‐analyses on the prevalence of IGD [16]; 6.8% boys and 1.3% girls [34]; 0.9%–8.5% total [46]; 6.3% male and 2.5% female), the DSM‐5 IGD diagnostic criteria identified a higher prevalence of IGD among boys (5.1%) compared to girls (0.0%) within the present cohort. The slight underestimation observed in the present cohort can be attributed to the influence of socio‐cultural aspects on the prevalence of online gaming and IGD. Existing research indicates that variables such as being raised in a single‐parent household [47] and lower levels of educational attainment [48] are associated with increased risk of these conditions. The present cohort predominantly comprised children of married (81.5%) and highly educated (60.1%) women, which may have contributed to the observed underestimation.

### 4.2 Gaming as a Secondary Symptom of pre‐existing Problems

A comparison between gaming and nongaming adolescents revealed no significant differences in mothers' reports of higher screen time or more emotional/behavioral symptoms by primary school age. It is assumed that other underlying mechanisms, such as contemporary media exposure and peer influences with less parental supervision during adolescence, play a role in online gaming during this developmental period. It has been concluded that online gaming has not been demonstrated to serve as a secondary symptom of earlier emotional or behavioral problems. However, it is imperative to note that these findings must be replicated, because our results represent the first longitudinal data on this topic. Nonetheless, a predictor has been identified that manifests during primary school age, when the focus is on gaming intensity rather than on the distinction between gaming and nongaming: Adolescent boys who played more excessively in terms of more DSM‐5 IGD symptoms demonstrated longer digital gaming screen times and more total symptoms during primary school age.

### 4.3 Gaming as a Risk‐Factor for Adolescent Current Mental Health

The data indicated that differences in mental health between gamers and nongamers were not yet evident at primary school age. Consequently, it is proposed that gaming is not a consequence of mental health problems, but may instead serve as a contributing factor. In this context, the findings of the present study suggest that online gamers exhibit elevated levels of self‐reported psychological stress and behavioral symptoms in comparison to nongamers. Furthermore, female adolescents demonstrated a higher prevalence of emotional symptoms. Additionally, an increased number of DSM‐5 IGD symptoms were associated with poorer self‐reports. This observation aligns with previous cross‐sectional results that have identified a link between gaming and mental health impairments in adolescents. These include higher rates of behavioral and emotional problems, diminished prosocial skills [49], and symptoms of depression, anxiety, ADHD, and obsessive‐compulsive disorder [18, 50, 51]. The present study is the first to thoroughly examine the relationship between elevated levels of DSM‐5 IGD symptoms and subsequent psychological stress. The present study demonstrated the aforementioned effect, a finding which is of particular relevance to clinical interventions.

Maternal reports on adolescent mental health outcomes exhibited no cross‐sectional association. This raises the question of how valid mothers' reports of adolescents' mental health symptoms are. The phenomenon of reduced maternal involvement, combined with the increasing autonomy of children during adolescence, appears to reduce maternal insight into their offspring's psychological status. Consequently, for IGD diagnostics, self‐reports regarding gaming may provide enhanced validity and a more effective means of identifying possible mental health consequences than third‐party interviews.

### 4.4 Boys and Girls Are at Different Risk

While girls reported experiencing emotional symptoms in relation to gaming and showed reduced HCCs when gaming scores were elevated, boys' gaming scores were primarily associated with behavioral symptoms and psychological stress. It is important to note that research utilizing HCC as a biomarker in the context of IGD remains scarce. A study involving a small cohort reported no alterations in HCC levels among IGD patients [52]. However, despite these findings, the authors concluded that alterations in the hypothalamic‐pituitary‐adrenal (HPA‐) axis are possible. The present study's findings of hypocortisolism in gaming girls with higher gaming scores could be indicative of HPA axis exhaustion/habituation resulting from repeated stress exposure, as described in previous works [53]. However, the current sample size is limited (*n* = 24), and the findings require replication. Differences in mental health outcomes between boys and girls may be due to differences in gaming habits. In previous studies, girls were found to predominantly engage in games centered on relationship maintenance and peer interaction. In comparison, boys preferred competitive gameplay [54] or games with depictions of violence [55, 56]. The findings reported herein, which show elevated levels of emotional symptoms in gaming girls and elevated behavioral symptoms in boys, are consistent with the findings of the aforementioned study.

### 4.5 Limitations and Future Works

The present sample consists of 178 mother‐adolescent dyads. However, some subcategories (e.g., *n* = 24 girls who play games) are underrepresented. Additionally, the educational attainment of the mothers and adolescents in our sample is notably high, possibly limiting the generalizability of the findings. Mothers and adolescents were asked about screen use and gaming behavior. To accurately assess screen time and gaming behavior, it is important to use additional objective measurement techniques, such as actimeters and appropriate software packages, on account of the potential for self‐report bias. The present study did not include an examination of characteristics of gaming behavior, such as game genre and time spent playing. This could be particularly important for games with violent content, as these games are hypothesized to increase behavioral symptoms [55, 56]. The cognitive correlates of online gaming have not been measured. However, previous studies have shown that video games can also have positive effects, such as improving cognitive skills like attention [57], visual processing [58], spatial sense [59], creativity [60] and problem solving skills [61]. Similarly, the present study failed to assess social aspects such as other real‐life leisure activities, despite the assumption that social components of online games are postulated to positively influence social skills in real‐life interactions [20, 62].

It is recommended that future studies examine larger cohorts, using objective technology‐based measures of gaming behavior and genre. In addition, positive effects of gaming, such as the development of cognitive and social skills, should be measured using outcome measures that incorporate cognitive tests and real‐life leisure activities, in addition to mental health outcomes.

## 5 Conclusion

The findings of this study demonstrate that online gaming among adolescents is associated with psychological stress, behavioral symptoms, and emotional problems in adolescence, with more gaming indicating a greater degree of impairment. In conclusion, online gaming poses significant health risks, and responsible use within reasonable limits and age‐appropriate access is essential. In the family setting, parental digital media use and family media regulations on adolescent gaming and social media use directly influence the prevention and treatment of IGD [63]. Previous findings suggest that a combination of autonomy‐providing and limit‐including monitoring strategies is effective in reducing digital media consumption among children and adolescents [64, 65]. Beyond the family setting, in the professional context, it is imperative that child and adolescent mental health professionals consider excessive online gaming as a potential risk factor for mental health disorders in adolescents. The implementation of widespread, child‐ and parent‐specific IGD awareness and prevention programs ‐ starting in primary school, with a focus on limiting screen time ‐ and a wide range of alternative leisure activities are important at all ages and for all genders.

## Author Contributions

**Stefan Mestermann:** investigation, formal analysis, writing – original draft, software, writing – review and editing, visualization, data curation. **Peter A. Fasching:** conceptualization, methodology, writing – review and editing, project administration, supervision, validation. **Matthias W. Beckmann:** conceptualization, methodology, writing – review and editing, project administration, supervision, validation. **Jonas Hemetsberger:** writing – original draft, writing – review and editing. **Oliver Kratz:** conceptualization, methodology, writing – review and editing, project administration, supervision, validation. **Gunther H. Moll:** conceptualization, methodology, writing – review and editing, project administration, supervision, validation. **Maximilian Bailer:** formal analysis, writing – review and editing. **Johannes Kornhuber:** conceptualization, investigation, methodology, writing – review and editing, project administration, supervision, validation. **Bernd Lenz:** conceptualization, methodology, writing – review and editing, supervision, validation. **Anna Eichler:** conceptualization, investigation, methodology, formal analysis, writing – original draft, software, writing – review and editing, project administration, supervision, data curation, validation, visualization.

## Ethics Statement

The study was approved by the Local Ethics Committee (4596 and 353_18B) and was conducted in accordance with the Declaration of Helsinki.

## Conflicts of Interest

The authors declare no conflicts of interest.

## Transparency Statement

The lead author Stefan Mestermann affirms that this manuscript is an honest, accurate, and transparent account of the study being reported; that no important aspects of the study have been omitted; and that any discrepancies from the study as planned (and, if relevant, registered) have been explained.

## Data Availability

The data that support the findings of this study are available on request from the corresponding author. The data are not publicly available due to privacy or ethical restrictions.
